# Prescribing trends of glaucoma drugs in six major cities of China from 2013 to 2017

**DOI:** 10.1371/journal.pone.0227595

**Published:** 2020-01-13

**Authors:** Lingyan Yu, Kai Ding, Lifang Luo, Zhenwei Yu

**Affiliations:** 1 Department of Pharmacy, Second Affiliated Hospital, College of Medicine, Zhejiang University, Hangzhou, Zhejiang, China; 2 Department of Pharmacy, Sir Run Run Shaw Hospital, College of Medicine, Zhejiang University, Hangzhou, Zhejiang, China; Mahidol University, THAILAND

## Abstract

**Objective:**

To evaluate the prescribing trends of glaucoma drugs in six major cities of China from 2013 to 2017.

**Methods:**

A descriptive analysis using pharmacy prescription data was conducted. Outpatient prescription data was extracted from the Hospital Prescription Analysis Cooperative Project. Prescribing patterns, trends of visits, and corresponding expenditures for glaucoma medications were analyzed.

**Results:**

A total of 84297 ambulatory prescriptions were included in the current study. Visits by glaucoma patients increased from 13808 in 2013 to 20060 in 2017. Over the same period, the yearly expenditure for glaucoma drugs increased from 2.33 million to 3.95 million Chinese Yuan (CNY). Among all the six classes of glaucoma drugs (prostaglandin analogues, carbonic anhydrase inhibitors, α-receptor agonists, β-receptor antagonists, cholinergic agonists and fixed combinations), β-receptor antagonists were the most commonly prescribed in 2013, accounting for 34.3% of patients, but gradually decreased to 27.1% in 2017. Prostaglandin analogues became the most frequently prescribed drugs in 2017, accounting for 30.2% of the visits. Prostaglandin analogues are the most expensive and yielded a total expenditure of 2.34 million CNY in 2017, followed by carbonic anhydrase inhibitors, α-receptor agonists, β-receptor antagonists, fixed combinations, and cholinergic agonists. Combination therapy became increasingly prescribed in 2017.

**Conclusion:**

Glaucoma prescribing practices exhibited substantial changes over the study period. The number of glaucoma prescriptions continuously increased from 2013 to 2017, leading to increased prescription costs. These findings implied a similar trend observed in previous studies, as well as recommendations in the appropriate guidelines.

## Introduction

Glaucoma is a group of progressive optic neuropathies that can lead to severe visual field loss and irreversible blindness if left untreated [1]. Glaucoma affects more than 70 million people worldwide, approximately 10% of which are bilaterally blind [2]. In China, 13.12 million people had glaucoma in 2015. As society is rapidly aging, this number is predicted to reach 25.16 million by 2050 [3]. Therefore, greater attention should been paid to the treatment of glaucoma.

Lowering of intraocular pressure is the only proven method for glaucoma treatment [4]. Although both laser therapy and surgery are available, medical therapy is the initial treatment option for the vast majority of patients [5]. Currently, there are six main categories of glaucoma medications including prostaglandin analogues (PG), β-receptor antagonists (BRA), carbonic anhydrase inhibitors (CAI), α-receptor agonists, cholinergic agonists, and fixed combinations [5–6].

Many factors should be considered with regard to the choice of drugs, such as the patient’s intraocular pressure, visual field, degree of fundus damage, dosing schedules, treatment adherence, cost, and adverse effects [7]. However, few studies have investigated the prescribing patterns and trends of glaucoma medications in China. This research aimed to explore the changes in prescription patterns of glaucoma and related expenditures by using a large sample of outpatients with glaucoma over a 5-year period from 2013 to 2017.

## Methods

### Study design

This research was designed as a retrospective descriptive study based on prescription data. The study was approved by the ethics committee of Sir Run Run Shaw Hospital, College of Medicine, Zhejiang University (20190628–22). Informed consent was waived by the same committee as part of the approval.

### Data source

Prescription data was extracted from the database of Hospital Prescription Analysis Cooperative Project. The objective of the project was to analyze prescription data of hospitals in China. Participating hospitals provided data on prescriptions to the research group for each sample day. There were forty randomized sampling days per year, including ten sampling days for each quarter. Prescription data included patients’ code, sex, age, date, and diagnosis, as well as drug generic name and price of the drug. The database for this project has been widely used [8–9].

In this study, prescription data of 56 hospitals in Beijing, Hangzhou, Chengdu, Guangzhou, Shanghai, and Tianjin were selected because these hospitals participated in the program continuously from 2013 to 2017, and were respectively located in the north, west, south, and east, thus covering a wide area of China. Brief hospital information is shown in S1 Table.

### Prescription inclusion and data extraction

Prescriptions containing at least one glaucoma drug for outpatients who had a diagnosis of glaucoma were included in this study. There is no restriction regarding the diagnostic criteria or the type or the severity of glaucoma. The study period was from 2013 to 2017. Prescriptions for patients aged below 18 were excluded. The following fields of prescriptions will be extracted: patient’s code, sex, age, date, location, and diagnosis; generic name and price of glaucoma drugs. Prescriptions with missing fields were excluded. Patient codes were reorganized by the dataset such that individual participants could not be identified.

This study was conduct between Jul 2019 and Sep 2019.

### Drug classes

Glaucoma drugs used in this study were classified into the following categories: PG, BRA, CAI, α-receptor agonists, cholinergic agonists and fixed combinations. Fixed combinations drops included bimatoprost with timolol and brinzolamide with timolol.

### Analysis

Primary analysis units of this study were treatment visits and expenditure of patients who were prescribed glaucoma drugs. A visit was defined as a prescription containing glaucoma drugs to a glaucoma patients, regardless if the prescription was new or for a refill. Expenditure was obtained by adding up all the prices of glaucoma drugs. Cost per visit, also called average cost, was calculated by dividing the total expenditure by the number of visits. Overall trends of glaucoma drug prescribing were described over the 5-year observation period. Trends for each class and some specific glaucoma drugs were also evaluated. Prescribing patterns were further analyzed by monotherapy and combination therapy. The rank-sum test was used to test the statistical significance of overall trends for visits and expenditure. The Cochran-Armitage trend test was applied to assess the statistical significance of prescribing trends of drugs and drug classes. All statistical analyses were conducted using R V.3.3.0 (http://www.R-project.org). A P value of < 0.05 was considered as statistically significant.

## Results

### Characteristics of included prescriptions

A total of 85255 prescriptions for outpatients who diagnosed with glaucoma were extracted. Of these, 84927 met the inclusion criteria and were included in this study. Table 1 shows the demographic characteristics of the populations of included prescriptions. The proportion of females (51.5%) was generally higher than male patients. The population with glaucoma aged below 40 was small, around 8% each year. Gender and age distributions for each year remained constant.

**Table 1:** The population demographic characteristics of included prescriptions from 2013 to 2017.

Year	2013	2013	2013	2013	2013
Age					
18–39	1076 (7.79)	1076 (7.79)	1076 (7.79)	1076 (7.79)	1076 (7.79)
40–49	1134 (8.21)	1134 (8.21)	1134 (8.21)	1134 (8.21)	1134 (8.21)
50–59	2324 (16.83)	2324 (16.83)	2324 (16.83)	2324 (16.83)	2324 (16.83)
60–69	3775 (27.34)	3775 (27.34)	3775 (27.34)	3775 (27.34)	3775 (27.34)
70 up	5499 (39.82)	5499 (39.82)	5499 (39.82)	5499 (39.82)	5499 (39.82)
Sex					
Male	8900 (46.12)	9677 (46.67)	9856 (45.85)	12408 (46.39)	12894 (45.84)
Female	10397 (53.88)	11057 (53.33)	11642 (54.15)	14342 (53.61)	15237 (54.16)

Data are represented as number of patients (%).

### Overall trends of glaucoma drug prescribing

**Fig 1:**
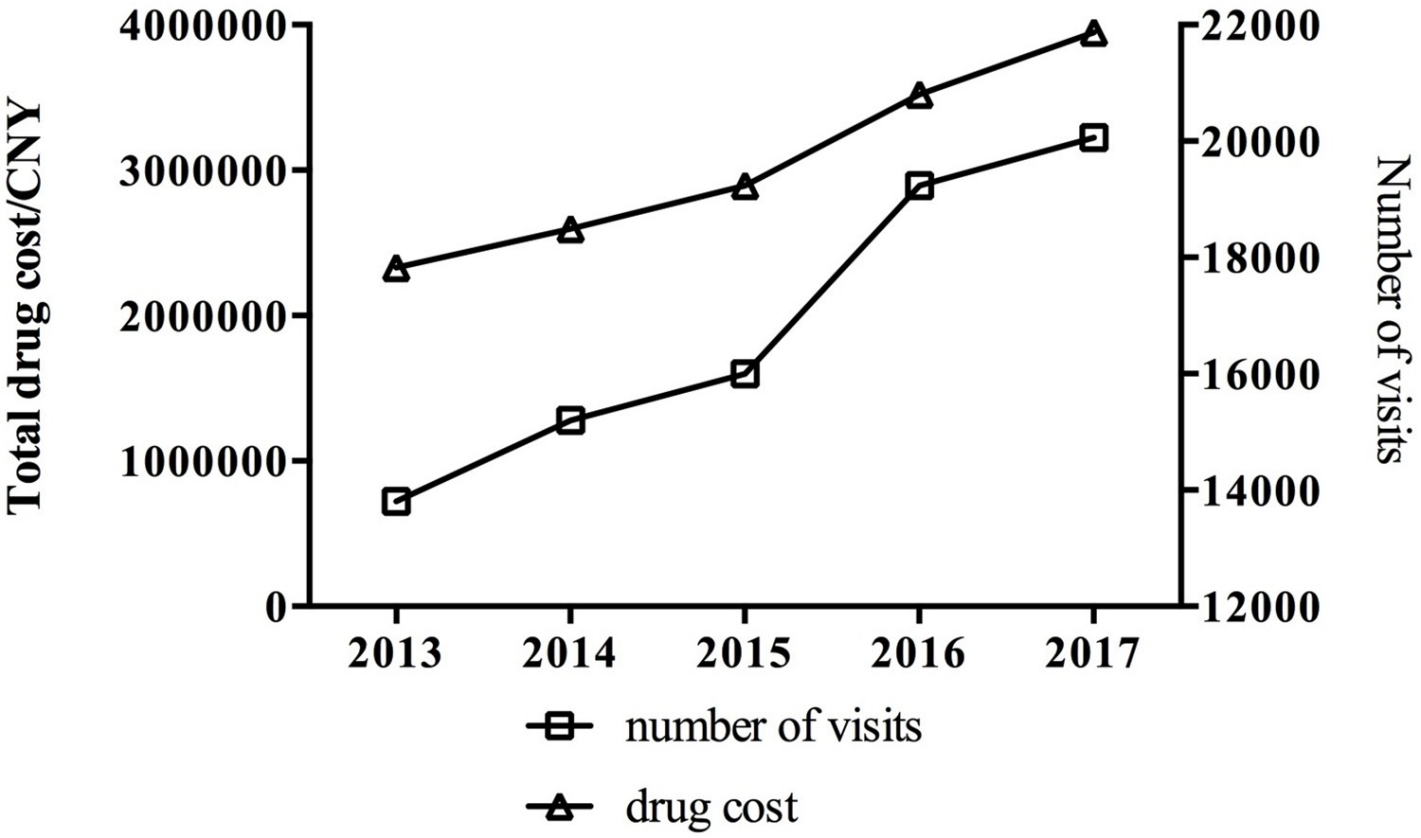
Total visits and cost trends of glaucoma drugs from 2013 to 2017.

Overall trends of glaucoma drug prescribing were described using visit and expenditure data. Our results showed that both visits and expenditure of glaucoma drugs increased during the observation period (both P < 0.05). As indicated in Fig 1, yearly visits of glaucoma patients had increased substantially from 13808 in 2013 to 20060 in 2017, representing a 45.3% increase over the study period. Meanwhile, a 69.5% increase in prescribing cost was found over the the same period, from 2.33 million Chinese Yuan (CNY) in 2013 to 3.95 million CNY in 2017. Trends for different geographical religions were shown in S1 Fig.

### Trends by drug class

**Fig 2:**
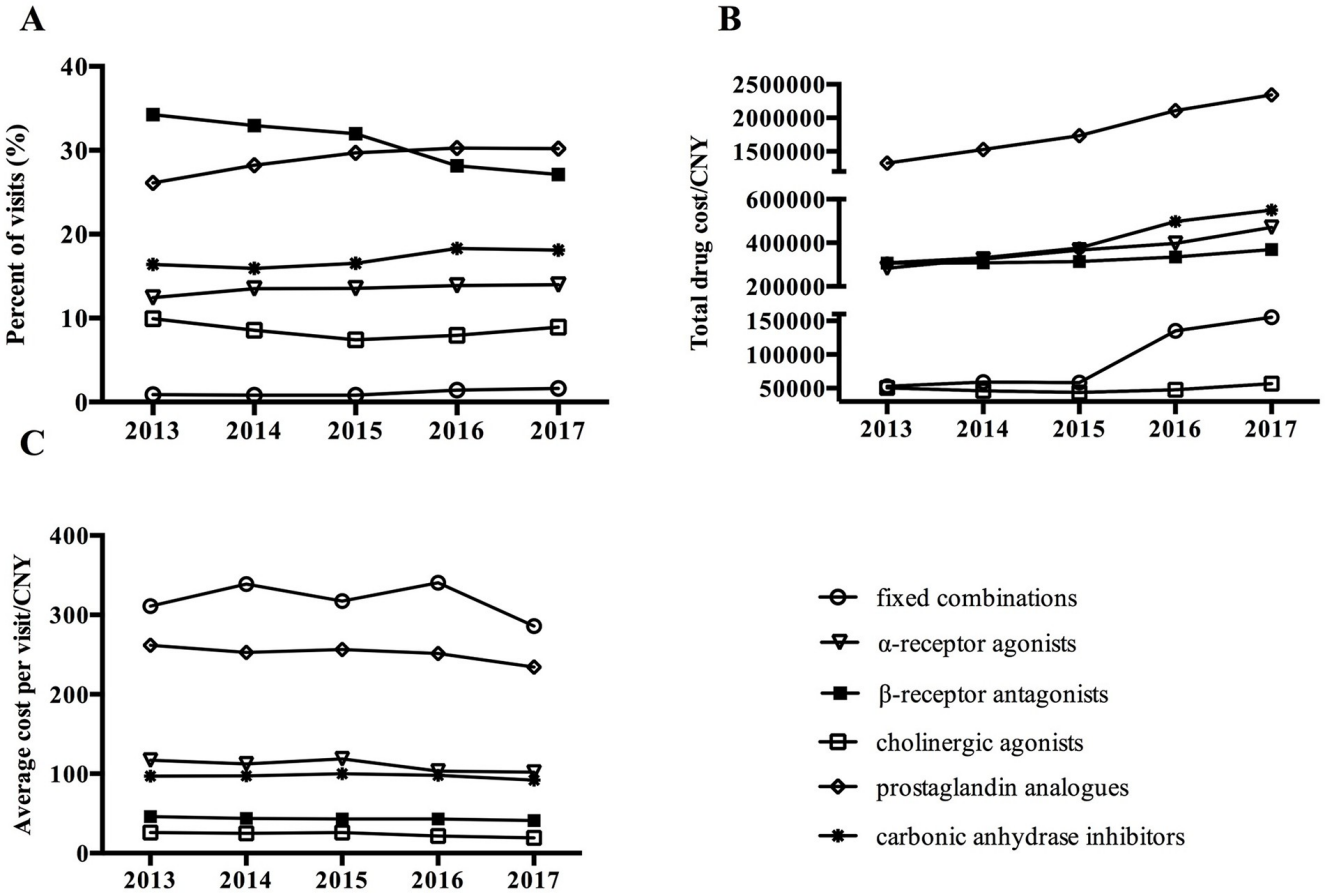
Prescriptions trends of glaucoma medications by drug class. (A) Trends in visit proportion; (B) Trends in expenditures; (C) Trends of average cost per visit.

Prescription trends of glaucoma drugs were further analyzed by drug class. Fig 2 shows trends of the visits and expenditures for the six class of glaucoma drugs. In 2013, BRA were prescribed for 34.3% of patients, making them the most commonly prescribed glaucoma drug that year. However, the percentage of BRA gradually decreased to 27.1% in 2017 (P < 0.05). PG became the most frequently prescribed glaucoma drug class in 2017, accounting for 30.21% of the visits (P < 0.05). Percentages of visits of CAI, α-receptor agonists, and cholinergic agonists all increased from 2013 to 2017, to a small but statistically significant extent (all P < 0.05). Fixed combinations were rarely prescribed at the beginning of the study, but their use increased rapidly in recent years. The percentage of visits with prescription of fixed combinations was 0.875% in 2013, increasing to 1.64% in 2017.

The increase in visits with PG prescriptions was also reflected in the corresponding expenditures during the study period. In 2017, 2.34 million CNY was spent on PG, making it the glaucoma drug class with the highest expenditure, followed by CAI, α-receptor agonists, BRA, fixed combinations, and cholinergic agonists (Fig 2B).

The average cost of each glaucoma drug class remained constant during the study period (Fig 2C). According to cost per visit, PG, the most frequently prescribed glaucoma drug class at the end of the study, were also the most expensive except for fixed combinations. BRA, the former most-popular class, had a much lower average cost compared with PG.

### Trends of specific drugs in top classes

**Fig 3:**
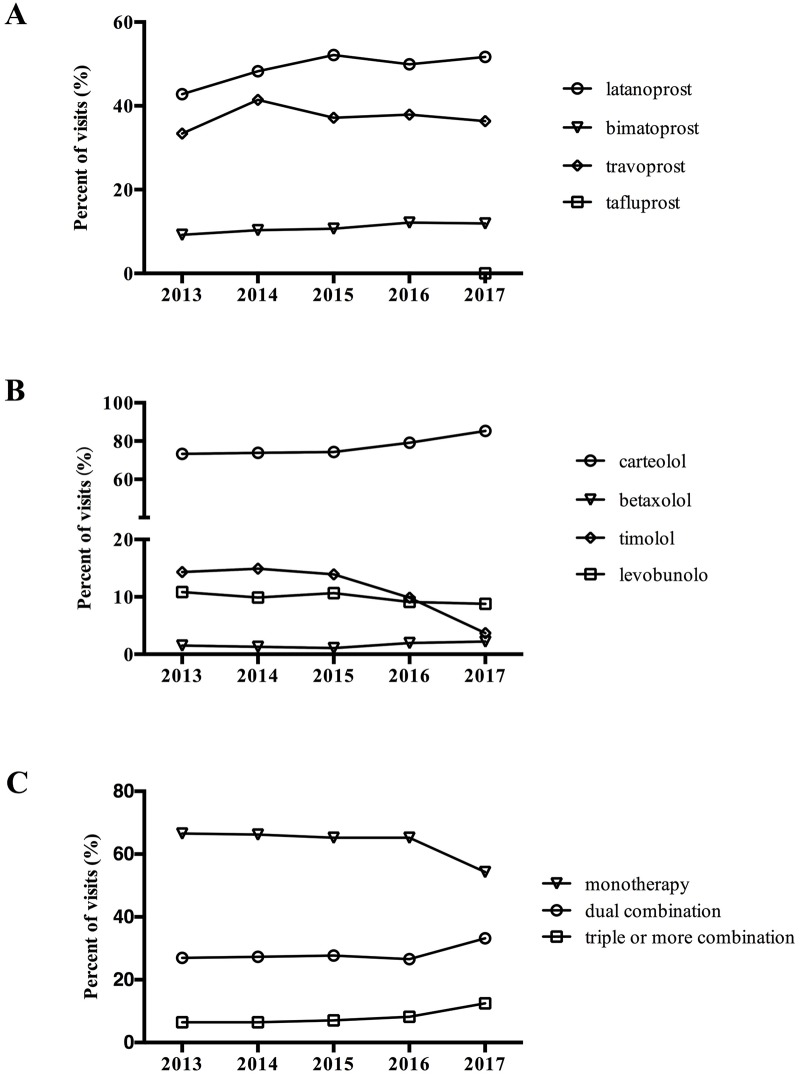
Trends in visits of specific classes of glaucoma drugs and prescribing patterns from 2013 to 2017. (A) Trends of prostaglandin analogues; (B) Trends of β-receptor antagonists; (C) Trends in the prescribing patterns of glaucoma drugs.

Prescribing trends of each drug in PG and BRA classes were analyzed by visits. The results shown in Fig 3A indicated that latanoprost was the most frequently prescribed PG, comprising almost 50% of all PG prescribed in 2017. Use of tafluprost, a new type of PG, was only observed in 2017. Carteolol was the most often used BRA, contributing nearly 85.3% of all BRA prescribed in 2017 (Fig 3B). Although use of carteolol continuously increased during the study period, this was not significant (P = 0.08). Notably, the use of timolol rapidly decreased (P < 0.05).

### Trends of prescribing patterns

Drugs with different mechanisms can be used in combination for patients whose glaucoma cannot be controlled by monotherapy. Trends of prescription patterns for monotherapy, dual combination, and triple-or-more combination are shown in Fig 3C. The percentage of combination medication was constant from 2013 to 2016, but increased in 2017. Percentages of dual combination prescriptions varied from 27.0% to 33.2% over the 5-year observation period. During this time, proportions of triple-or-more combination prescriptions dramatically increased from 6.47% to 12.49%.

## Discussion

Prescriptions trends of glaucoma medications in China have been revealed for the first time. We found that both the number of patients receiving glaucoma drugs and the total cost increased rapidly during the observation period. Moreover, the preferred drug had been changed and combination therapy became a more favorable option.

Prescription of glaucoma medications showed an upward trend between 2013 and 2017, which was basically consistent with previous reports in the literature [10]. An increasing number of patients with glaucoma was also revealed in another study [3]. Although glaucoma can occur at any age, aging is a major risk factor [11–12]. The prolonged life expectancy of the Chinese population and a rapidly aging society result in an increased number of elderly individuals with a high risk of glaucoma [13]. Our results showed that the most patients were aged above 40 years, consistent with previous studies [14–15]. Increased numbers of glaucoma patients led to an increase in medical expenditure; in particular, increased acquisition of PG, which had a higher cost, contributed to the incremental increase in total expenditure.

PG and BRA accounted for a large proportion of prescriptions during the study period, which may be related to the characteristics of drug action, price, and regional economic level. The major trend observed was the conversion of BRA prescriptions into PG prescriptions. PG, which is utilized to increase uveoscleral outflow and decrease aqueous humor production, effectively lowers intraocular pressure and also acts during the night [16]. Some studies have demonstrated PG to be more efficacious than BRA with fewer systemic side effects [17–18]. Notably, the consistency of treatment adherence in patients may be improved by its once-daily administration [19]. In addition, many guidelines recommended use of PG as the first-line medication for most patients since latanoprost was introduced in the United States as a new chemical entity for the topical treatment of glaucoma [20–21]. We predicted that use of PG will increase in accordance with previous researches that found PG to be dominant for the treatment of glaucoma in Europe and North America [22–24]. Indeed, expenditures of PG significantly increased from 2013 to 2017. Notably, although increasing expenditure put greater pressure on the health care system, few pharmacoeconomic studies have investigated the cost-effectiveness of prostaglandin, especially in China.

BRA were usually prescribed as the main medication for glaucoma patients [25]. BRA are favored by ophthalmologists because of their low price and large antihypertensive range. Moreover, guidelines also recommend BRA as alternatives to PG for patients because of unavailability, cost issues, or possible adverse reactions [5]. Use of BRA in China gradually decreased during the study period, but it still played an important role in the treatment of glaucoma. There are some important side effects of these drugs, such as the slowing down of heart rate and bronchospasm; thus, and attention should be paid to the management of side effects and patient compliance [26].

Despite CAI not being the first-line drug recommended by the guidelines for lowering intraocular pressure in glaucoma, the proportion of CAI prescriptions has been nearly 16% in recent years. The mechanism by which CAI works is to reduce aqueous humor production by inhibiting carbonic anhydrase [6]. In addition, use of α-receptor agonists, which provide a certain protective effect on the optic nerve, was almost 13% in this study. Cholinergic agonists are no longer commonly used for the treatment of open-angle glaucoma or ocular hypertension, mainly because of poor tolerance to the side effects of these drugs. According to the National Institute for Clinical Excellence (NICE) guideline, the first choice for glaucoma should be PG, and if these are not tolerated, a BRA should be offered. If none of these options is tolerated, offering CAI, α-receptor agonists, and cholinergic agonists, or a combination of treatments should be considered [5]. Clinical evidence indicates no clinical difference between these drugs, but CAI are better tolerated than α-receptor agonists[27].

Trend analysis of combined drug use identified that the proportion of combined drug use has increased in recent years. Ideally, the target intraocular pressure should be achieved with the fewest medications and minimum adverse effects. According to guidelines, if monotherapy can not achieve the target intraocular pressure, combined drug therapy with different mechanisms could be chosen [5]. The Collaborative Initial Glaucoma Treatment Study found that more than one single class of medications is needed for the many patients [28]. Indeed, combined use of two or more drugs can enhance the clinical therapeutic effect by different mechanisms, as well as improve patient comfort.

The observed changes in prescription practices may also be the result of factors such as the approval and availability of new drugs, physicians prescribing habits, and pharmaceutical advertisements. Thus, the offering of any glaucoma medication, contraindications, allergies, comorbidities, and drug interactions should be considered.

This study has several limitations. First, important patient information such as severity of glaucomatous optic neuropathy, visual field defect, and other glaucoma-related parameters was not acquired. Moreover, outcomes of pharmacological therapy were also not obtained. As included hospitals were located in major cities, sampling bias may have occcured. In addition, the burden of glaucoma, rather than drug cost, should be fully investigated in future studies.

## Conclusion

Glaucoma drug prescribing was found to increase with regard to both patient visits and drug expenditure in the current study. Prescribing practices exhibited substantial changes over the 5-year study period. Drugs with proven efficacy and guideline recommendations were preferred, although their prices were relatively higher. However, as growing expenditures put increasing pressure on the health care system and patients, pharmacoeconomic studies evaluating the cost-effectiveness and cost-utility of glaucoma drugs are needed.

## Supporting information

S1 Fig Visits and cost trends of glaucoma drugs in different geographical religions. (TIFF)

S1 Table Brief hospital information of included hospitals. (PDF)
