# Correction: Prescribing trends of glaucoma drugs in six major cities of China from 2013 to 2017

**DOI:** 10.1371/journal.pone.0230694

**Published:** 2020-03-13

**Authors:** Lingyan Yu, Kai Ding, Lifang Luo, Zhenwei Yu

There are a number of errors in the Year and Age values of Table 1. Please see the correct Table 1 here.

**Table 1:** The population demographic characteristics of included prescriptions from 2013 to 2017.

Year	2013	2014	2015	2016	2017
Age					
18–39	1076 (7.79)	1196 (7.87)	1263 (7.90)	1648 (8.57)	1694 (8.44)
40–49	1134 (8.21)	1197 (7.88)	1304 (8.15)	1604(8.34)	1794 (8.94)
50–59	2324 (16.83)	2463 (16.20)	2439 (15.25)	3035 (15.78)	3139 (15.65)
60–69	3775 (27.34)	4156 (27.34)	4557(28.49)	5524 (28.72)	5828 (29.05)
70 up	5499 (39.82)	6188 (40.71)	6434 (40.22)	7421 (38.59)	7605 (37.91)
Sex					
Male	8900 (46.12)	9677 (46.67)	9856 (45.85)	12408 (46.39)	12894 (45.84)
Female	10397 (53.88)	11057 (53.33)	11642 (54.15)	14342 (53.61)	15237 (54.16)

Data are represented as number of patients (%).
