# Supplementary figures and images for: Prescribing trends of glaucoma drugs in six major cities of China from 2013 to 2017
Source: PLoS One. 2020 Jan 13;15(1):e0227595. doi: 10.1371/journal.pone.0227595 (PMC6957137; doi:10.1371/journal.pone.0227595)

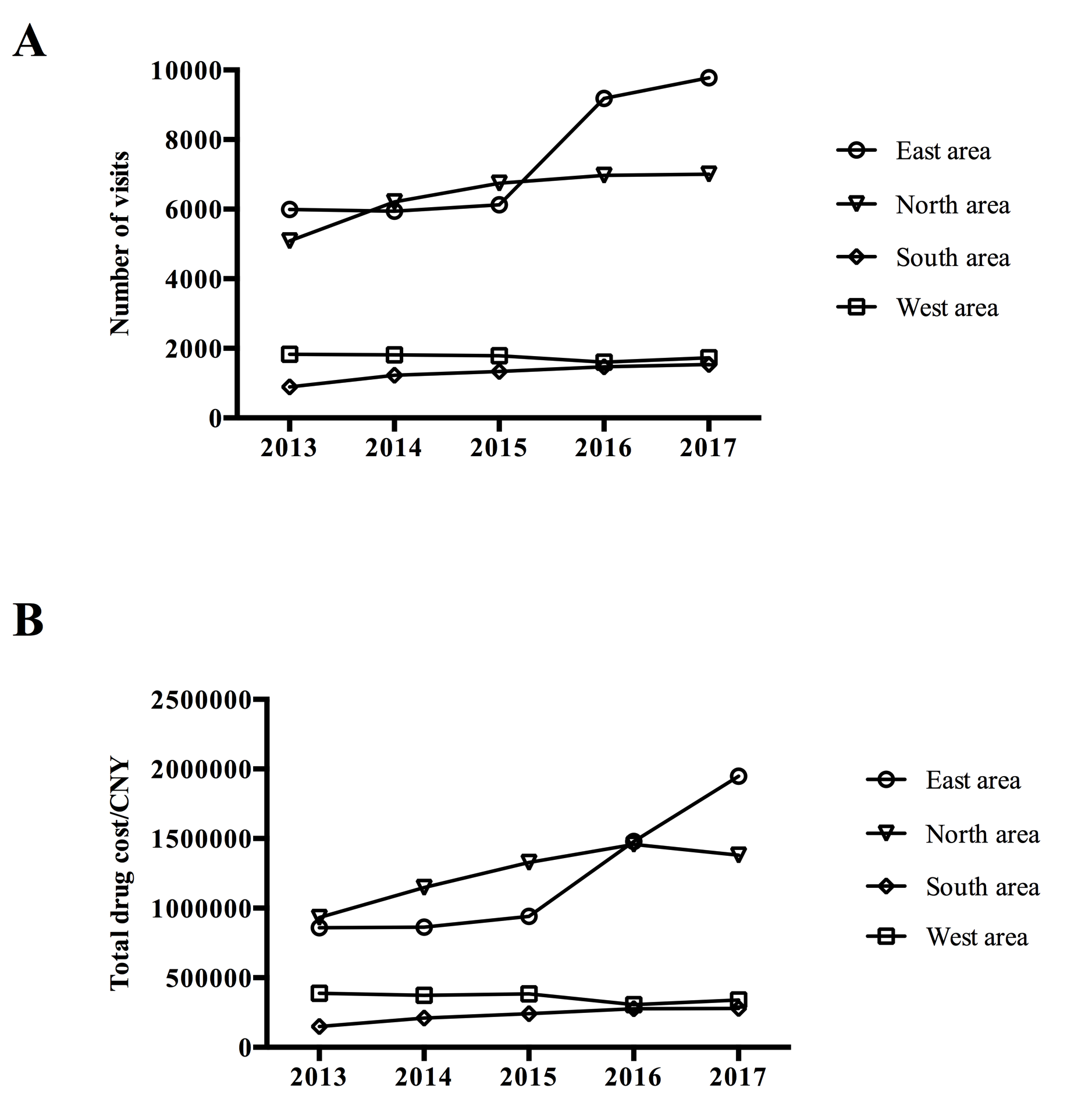

Supplement: S1 Fig — (TIFF) [file pone.0227595.s001.tiff]
