# Supplementary material for: Prescribing trends of glaucoma drugs in six major cities of China from 2013 to 2017
Source: PLoS One. 2020 Jan 13;15(1):e0227595. doi: 10.1371/journal.pone.0227595 (PMC6957137; doi:10.1371/journal.pone.0227595)
Supplement: S1 Table — (PDF) [file pone.0227595.s002.pdf]

S1 Table. Brief hospital information of included hospitals.

Religion	Cities	Hospital description
East area	Shanghai	11 tertiary hospitals, 5 secondary hospitals, all state owned
	Hangzhou	9 tertiary hospitals, all state owned
North area	Beijing	9 tertiary hospitals, 2 secondary hospitals, all state owned
	Tianjing	6 tertiary hospitals, 1 secondary hospital, all state owned
West area	Chengdu	6 tertiary hospitals, all state owned
South area	Guangzhou	7 tertiary hospitals, all state owned
